# Increased survival after irradiation followed by regeneration of bone marrow stromal cells with a novel thiol-based radioprotector

**DOI:** 10.3325/cmj.2014.55.45

**Published:** 2014-02

**Authors:** Ivana Okić-Djordjević, Drenka Trivanović, Miloš Jovanović, Marija Ignjatović, Bojana Šećerov, Miloš Mojović, Diana Bugarski, Goran Bačić, Pavle R. Andjus

**Affiliations:** 1 Institute for Medical Research, University of Belgrade, Belgrade, Serbia; 2 Faculty of Biology, University of Belgrade, Belgrade, Serbia; 3 Vinča Institute of Nuclear Sciences, University of Belgrade, Belgrade, Serbia; 4 Faculty of Physical Chemistry, University of Belgrade, Belgrade, Serbia

## Abstract

**Aim:**

To investigate the survival of laboratory rats after irradiation and to study the cellularity of their bone marrow and the multipotential mesenchymal stem cells (BM-MSCs) in groups treated with or without a new thiol-based radioprotector (GM2011)

**Methods:**

Animals were irradiated by a Cobalt gamma source at 6.7 Gy. Treated animals were given i.p. GM2011 30 minutes before and 3 and 7 hours after irradiation. Controls consisted of sham irradiated animals without treatment and animals treated without irradiation. After 30 days post-irradiation, animals were sacrificed and bone marrow cells were prepared from isolated femurs. A colony forming unit-fibroblast (CFU-F) assay was performed to obtain the number of BM-MSCs.

**Results:**

In the treated group, 87% of animals survived, compared to only 30% in the non-treated irradiated group. Irradiation induced significant changes in the bone marrow of the treated rats (total bone marrow cellularity was reduced by ~ 60% – from 63 to 28 cells ×10^6^/femur and the frequency of the CFU-F per femur by ~ 70% – from 357 to 97), however GL2011 almost completely prevented the suppressive effect observed on day 30 post-irradiation (71 cells ×10^6^/femur and 230 CFU-F/femur).

**Conclusion:**

Although the irradiation dosage was relatively high, GL2011 acted as a very effective new radioprotector. The recovery of the BN-MSCs and their counts support the effectiveness of the studied radioprotector.

Radiotherapy is common in cancer treatment and around 80% of cancer patients need radiotherapy either for curative or palliative purposes (1). Despite of the recent development of sophisticated radiotherapeutic machines and protocols (eg, conformal therapy, multileaf collimators, etc) designed to focus therapeutic beams on the tumor, irradiation of adjacent healthy tissue is unavoidable, thus limiting therapeutic gain (2). One potential way to alleviate the problem is the use of radioprotectors that will reduce the deleterious effect of ionizing radiation on healthy tissue. For decades, literally thousands of radioprotectors (most of them were free radical scavengers) have been tested (2,3) but surprisingly only a few of them are in use today. In fact, the only radioprotective/cytoprotective agent specifically approved by the Food and Drug Administration is phosphorotioate amifostine (WR-2721), which is in effect a scavenger of highly reactive free radicals induced by radiation, but also might have some other mechanism of action (4). However, the use of amifostine has some drawbacks since it has been found that it produces undesirable side effects (hypotension, vomiting, hot flashes, hypocalcemia, etc) (5,6). These can be quite severe, thus limiting the amount of the drug that can be administered to levels lower than necessary to achieve maximal radioprotection. Therefore, the use of amifostine is limited to controlled clinical situations. Moreover, it is effective only if administered prior to irradiation and cannot be applied to protect in cases of exposures to radiation (eg, as in space missions or in accidents in nuclear plants) (7). Consequently, the search for novel, non-toxic and convenient radioprotectors is still on (8-14).

In this article, we studied a newly synthesized, naturally occurring sulfur-containing aminothiol compound, GL2011, previously demonstrated to be non-toxic to rodents (behavioral and gastrointestinal tests, unpublished), for its radioprotective capabilities upon total body irradiation. The essential criterion in testing any drug as a potential radioprotector is a 30-day survival of animals irradiated at doses close to LD_50_. Along with that, numerous other parameters have been used at the endpoint or during these 30 days to access the action of radioprotectors. These include DNA damage, membrane lipid peroxidation, tissue morphology, etc, but most studies focused on hematopoietic and gastrointestinal systems. The best protocol would be to sacrifice and analyze animals at different time intervals post-irradiation since follow-up during survival would be invasive. Thus, a marker is needed to show the difference between radioprotected and those animals that survive radiation. In this initial phase study, we chose to use as a radioprotection marker the state of bone marrow multipotential mesenchymal stem cells or bone marrow stromal cells (BM-MSCs), estimated by their clonogenic potential and quantified by Colony Forming Unit-Fibroblast (CFU-F) assay. BM-MSCs are essential in providing support for the growth and differentiation of primitive hemopoietic cells within the bone marrow microenvironment. BM-MSCs have also been shown to contribute to the regeneration of different mesenchymal tissues, and therefore generated a great deal of interest in many clinical settings, including that of regenerative medicine, immune modulation, and tissue engineering. These cells thus have a substantial value for the survival and recovery after radiation and are chosen as a relevant radioprotective marker.

## Materials and methods

### Animals

The experiments were performed on male albino Wistar rats weighting 200 g (±5%). Animals (2 per cage) were housed prior and after irradiation at ambient temperature (20-23°C), 12-hour light/dark intervals. Food and water were given *ad libitum*.

Animals were randomized into groups as follows (30 per group):

Group I (irradiated with GL2011 treatment) – animals were treated with GL2011 at standard time intervals: -30 minutes (prior to irradiation), 3 hours and 7 hours (following the irradiation) intraperitoneally (i.p.), 100 mg/kg of body weight (minimal dose selected based on unpublished preliminary studies), 1 mL per injection, with GL dissolved in phosphate-buffered saline (PBS).

Group II (radiation control – irradiated without GL2011 treatment) – animals were i.p. injected with PBS at -30 minutes (prior to irradiation), 3 hours and 7 hours (following the irradiation).

Group III (drug control – no irradiation) i.p. injected with GL2011, the same dose and time schedule as for the group I.

Group IV (sham – no irradiation, no drug treatment) – injected with PBS at intervals as before.

Following irradiations, animals were inspected twice a day (morning and evening) and moribund animals were euthanized. Animals surviving day 30 after irradiation were sacrificed by guillotine and their femurs were removed for processing and subsequent cell preparation.

All experiments were performed in fall and winter of 2011 at the Vinča Institute of Nuclear Sciences and the Institute of Medial Research both at the University of Belgrade and were approved by the Committee for Ethical Animal Care and Use of the Faculty of Biology, Belgrade (approval no 04/2012, 27/05/2012), which acts in accordance with the Guide for the Care and Use of Laboratory Animals published by the US National Institute of Health (NIH Publication No. 85/25, reviewed in 1986).

### Irradiation

Cobalt gamma source designed for radiobiological and radiation chemistry experiments (Vinča Institute of Nuclear Sciences, Belgrade) was used for irradiation. Calibration of the source for these experiments was performed by measuring midline absorbed doses in agarose gel phantoms of a rat with embedded plastic vials containing Fricke solution, and doses were determined by spectrophotometry. Based on literature data, the dose of 6.7 Gy was selected as a potential LD_50_ dose at 30 days post-irradiation (LD_50/30_) (8,11,15,16). Unanesthetized animals were confined in custom made individual cages made of wire. Total body irradiation was performed with rats sideways to the source at the dose rate of 0.41 Gy/min, ie, 16 minutes of irradiation for the dose of 6.7 Gy.

### Cell preparation

Bone marrow cells were flushed out of the femurs with DMEM medium and single-cell suspension was prepared from each animal in Dulbecco’s modified Eagle’s medium (PAA Laboratories, Yeovil, UK) supplemented with 10% fetal calf serum (PAA Laboratories).

*Bone marrow cellularity and viability* – The total number of nucleated cells in bone marrow was enumerated in hemocytometer using Türck staining solution and the viability of the cells was determined by trypan blue exclusion test.

*Colony forming unit-fibroblast (CFU-F) assay* – CFU-F assays were performed by plating 1 × 10^6^, 1 × 10^7^_,_ and 2 × 10^7^ bone marrow cells/well in 9.5 cm^2^ cell culture dishes (6-well plate; Sarstedt, Numbrecht, Germany) in duplicates. After 8 days in culture at 37°C in a humidified atmosphere containing 5% CO_2_, the cells were washed, fixed in ice-cold methanol for 4 minutes, and stained with Giemsa for 10 minutes. The number of CFU-Fs was determined by counting the number of visible colonies.

Statistical analysis of the survival data was performed by Kaplan-Meier Survival analysis and data proportions were compared by the two-tailed Fisher exact test, while mean survival times and cell colonies counts were compared by *t* test.

## Results and discussion

### Irradiaton

The mortality of non-protected animals was highest between days 10-20 after irradiation, which is in accordance with other studies of the same type (8,10,11,16,17) (Table 1). The animals that were treated with the radioprotector GL2011 but still did not survive reached the moribund state between days 11-15. No apparent effects of GL2011 on the appearance and behavior of animals were noticed.

**Table 1:** Results of 30-d survival studies and radioprortection by GL2011 in four equal groups (n = 30)

Group/treatment	Survival – n (%)	Mean survival time (days ± standard deviation)
Group I (irradiated treated)	26 (87%)	27 ± 6
Group II (irradiated nontreated)	9 (30%)	16 ± 11*
Group III (nonirradiated treated)	30 (100%)	>30
Group IV (nonirradiated nontreated)	30 (100%)	>30

*P < 0.001.

Our applied dose of 6.7 Gy was higher than the true LD_50/30,_ since only 30% of unprotected animals survived (Table 1). This could be the consequence of using younger animals (only 200 g b.w.) than in other studies, since they are more radiosensitive than older animals (18). Consequently, survival of 87% of animals can be considered as an excellent radioprotection. This is better than (or at least equal to) protection obtained in most animal studies using higher amifostine doses. Namely, Pamujula et al (17) demonstrated a 91% survival and mean survival time of 28 days in mice with 500 mg/kg amifostine, while the study of Trajkovic et al (10) on rats showed a survival of about 60% at the 30th day post-irradiation with 300 mg/kg.

### Bone marrow cellularity and CFU-F assay data

The irradiation without treatment induced significant changes in the bone marrow of the treated rats, since the total bone marrow cellularity was reduced by almost 60% and the frequency of the BM-MSCs per femur by about 70%, as compared to non-irradiated, control animals (Table 2, Group II vs IV). However, the application of the radioprotector GL2011 almost completely prevented the suppressive effect observed on day 30 post-irradiation. Namely, in respect to both the total number of bone marrow nucleated cells, as well as to the CFU-F number per femur, the values determined in the Group I, ie, the irradiated animals treated with GL2011, showed no significant difference from either the drug-treated only (Group III) or the non-irradied group without treatment (Group IV). The unchanged viability among groups at day 30 post-irradiation indicates the consistency of the isolation technique and confirms that GL2011 is specific and efficient for repairing the overall bone marrow cellularity, as well as the subpopulation of BM-MSCs

**Table 2:** The effect of radioprortection by GL2011 on the bone marrow cellularity and the frequency of bone marrow multipotential mesenchymal stem cells (estimated by Colony Forming Unit-Fibroblast assay, CFU-F)

Groups*	Cellularity ( × 10^6^/femur)^†^	Viability (%)	CFU-F/femur
Group I (irradiated treated)	71.5 ± 22.4^‡^	63.2 ± 13.3	230.2 ± 175.9^§^
Group II (irradiated nontreated)	28.1 ± 15.0	74.2 ± 19.3	97.1 ± 87.5
Group III (nonirradiated treated)	82.5 ± 20.9^‡^	79.3 ± 11.6	232.9 ± 123.1^§^
Group IV (nonirradiated nontreated)	62.9 ± 23.2^‡^	89.9 ± 7.6	356.6 ± 268.9^§^

*Experimental groups were the same as in Table 1.

†Data are presented as mean ± standard deviation.

‡*P* < 0.001, *t* test.

§*P* < 0.05, as compared to the Group II.

The BM-MSCs contain a high proliferative potential and ability for self-renewal and are essential for the regeneration of the hematopoietic system following total body irradiation. Although it has been previously shown that as compared to hematopoietic progenitors, mesenchymal progenitors are differentially sensitive to radiation (19), the mechanisms used by MSCs to survive radiation doses lethal to the hematopoietic system are poorly understood. Data reporting drug induced radioprotection indicated that the protective effects could be associated with the maturation stage, proliferation, and differentiation state or the alterations of the cell cycle of the progenitor cells (19,20). Our study demonstrated that the treatment with GL2011 provided almost complete recovery of the bone marrow cellularity and the frequency of the CFU-Fs 30 days post-irradiation. However, in order to differentiate the radioprotective from the regenerative role of GL2011 further studies are needed, both by exposing in vitro bone marrow cells to different irradiation doses, or by evaluating in vivo the recovery pattern of bone marrow tissue.

## References

[1] Steel GG. Basic clinical radiobiology. 3rd ed. London: Edward Arnold Publishers; 2002.

[2] Nair CKK, Parida DK, Namura T (2001). Radioprotectors in radiotherapy.. J Radiat Res (Tokyo).

[3] Weiss JF, Landauer MR (2003). Protection against ionizing radiation by antioxidant nutrients and phytochemicals.. Toxicology.

[4] Grdina DJ, Kataoka Y, Murley JS (2000). Amifostine: mechanisms of action underlying cytoprotection and chemioprevention.. Drug Metabol Drug Interact.

[5] Kligerman MM, Glover DJ, Turrisi AT, Norfleet AL, Yuhas JM, Coia LR (1984). Toxicity of WR-2721 administered in single and multiple doses. Int J Radiat Oncol Biol Phys.

[6] Koukourakis MI (2002). Amifostine in clinical oncology: current use and future applications.. Anticancer Drugs.

[7] Dziegielewski J, Goetz W, Baulch JE (2010). Heavy ions, radioprotectors and genomic instability: implications for human space exploration.. Radiat Environ Biophys.

[8] Sharma M, Kumar M (2007). Radioprotection of Swiss albino mice by *Myristica fragrans houtt.*. J Radiat Res (Tokyo).

[9] Shirazi A, Ghobadi G, Ghazi-Khansari M (2007). A radiobiological review on melantoin: a novel radioprotector.. J Radiat Res (Tokyo).

[10] Trajkovic S, Dobric S, Jacevic V, Dragojevic-Simic V, Milovanovic Z, Djordjevic A (2007). Tissue-protective effects of fullerenol C_60_(OH)_24_ and amifostine in irradiated rats.. Colloids Surf B Biointerfaces.

[11] Mihailovic M, Milosevic V, Grigorov L, Poznanovic G, Ivanovic-Matic S, Grdovic N (2009). The radioprotective effect of α_2_-macroglobulin: A morphplogical study of rat liver.. Med Sci Monit.

[12] Davis RM, Sowers AI, DeGraff W, Bernardo M, Thetford A, Krishna MC (2011). A novel nitroxide is an effective brain redox imaging contrast agent and *in vivo* radioprotector.. Free Radic Biol Med.

[13] Kunwar A, Jayakumar S, Bhilwade HN, Bag PP, Bhatt H, Chaubey RC (2011). Protective effects of Selenocystine against γ-radiation induced genotoxicity in Swiss albino mice. Radiat Environ Biophys.

[14] Kunwar A, Bag PP, Chattopadbyay S, Jain VK, Ptiyadarsini KI (2011). Anti-apoptotic, anti-inflammatory, and immunomodulatory activities of 3,3′-diselenodipropionic acid in mice exposed to whole body γ-radiation.. Arch Toxicol.

[15] Logie LC, Harris MD, Tatsch RE, van Hooser EN (1960). An analysis of the LD_50(30)_ as related to radiation intensity.. Radiat Res.

[16] Sevaljevic L, Dobric S, Bogojevic D, Petrovic M, Koricanac G, Vulovic M (2003). The radioprotective activities of turpentine-induced inflammation and α_2_-microglobulin: The effect of dexamethasone on the radioprotective efficacy of the inflammation.. J Radiat Res (Tokyo).

[17] Pamujula S, Kishore B, Rider B, Fermin CD, Graves RA, Agrawal KC (2005). Radioprotection in mice following oral delivery of amifostine nanoparticles.. Int J Radiat Biol.

[18] Jones DC, Osborn GK, Kimeldorf DJ (1969). Age at X-irradiatio and acute mortality in the adult male rat.. Radiat Res.

[19] Ramdas J, Warrier RP, Charles Scher C, Larussa V (2003). Effects of amifostine on clonogenic mesenchymal progenitors and hematopoietic progenitors exposed to radiation.. J Pediatr Hematol Oncol.

[20] Davis TA, Mungunsukh O, Zins S, Day RM, Landauer MR (2008). Genistein inducesradioprotection by hematopoietic stem cell quiescence.. Int J Radiat Biol.

